# Mechanism of Insoluble Aggregate Formation in a Reconstituted Solution of Spray-Dried Protein Powder

**DOI:** 10.1007/s11095-023-03524-x

**Published:** 2023-05-02

**Authors:** Yeqing Tao, Yuan Chen, Wesley Howard, Mariam Ibrahim, Sajal M. Patel, William P. McMahon, Yoen Joo Kim, Jared A. Delmar, Darryl Davis

**Affiliations:** 1 Process and Analytical Sciences, Biopharmaceuticals R&D, AstraZeneca, Gaithersburg, MD 20787 USA; 2 Dosage Form Design & Development, Biopharmaceuticals R&D, AstraZeneca, Gaithersburg, MD USA

**Keywords:** biophysical characterization, HDX-MS, particles, protein aggregation, spray-dry, stability

## Abstract

**Background:**

Spray-drying is considered a promising alternative drying method to lyophilization (freeze-drying) for therapeutic proteins. Particle counts in reconstituted solutions of dried solid dosage forms of biologic drug products are closely monitored to ensure product quality. We found that high levels of particles formed after reconstitution of protein powders that had been spray-dried under suboptimal conditions.

**Methods:**

Visible and subvisible particles were evaluated. Soluble proteins in solution before spray-drying and in the reconstituted solution of spray-dried powder were analyzed for their monomer content levels and melting temperatures. Insoluble particles were collected and analyzed by Fourier transform infrared microscopy (FTIR), and further analyzed with hydrogen-deuterium exchange (HDX).

**Results:**

Particles observed after reconstitution were shown not to be undissolved excipients. FTIR confirmed their identity as proteinaceous in nature. These particles were therefore considered to be insoluble protein aggregates, and HDX was applied to investigate the mechanism underlying aggregate formation. Heavy-chain complementarity-determining region 1 (CDR-1) in the aggregates showed significant protection by HDX, suggesting CDR-1 was critical for aggregate formation. In contrast, various regions became more conformationally dynamic globally, suggesting the aggregates have lost protein structural integrity and partially unfolded after spray-drying.

**Discussion:**

The spray-drying process could have disrupted the higher-order structure of proteins and exposed the hydrophobic residues in CDR-1 of the heavy chain, contributing to the formation of aggregate through hydrophobic interactions upon reconstitution of spray-dried powder. These results can contribute to efforts to design spray-dry resilient protein constructs and improve the robustness of the spray-drying process.

**Supplementary Information:**

The online version contains supplementary material available at 10.1007/s11095-023-03524-x.

## Introduction

The number of protein therapeutics reaching the market is growing rapidly each year. In contrast to small molecules, the highly complex structures of proteins make them more susceptible to changes throughout the manufacturing process. Protein aggregation is one of the most important critical quality attributes and can lead to decreased efficacy [1] and cause immunogenicity [2, 3]. Protein aggregation can be reversible self-association or effectively irreversible aggregation [3, 4],which have been extensively reviewed in the literature [5–8].

Aggregation pathways are highly dependent on protein properties. In general, protein aggregation in solution starts with protein-protein interactions, which may result from disruption of the native protein structure. Dimer or oligomers form initially, leading to subsequent growth in aggregate size and changes in morphology [3]. If this process continues, subvisible or visible particles eventually form, which may result in changes in product appearance and quality. The size of aggregates can range from several microns to nanometers [4]. The number and size of the particles can be measured with various optical measurements, depending on the size of the particles [4]. Thus, particle counts are closely monitored through the manufacturing process before release of the final drug product. Detailed requirements for parenteral drugs are specified in chapters 787 and 788 of the U.S. Pharmacopeia (USP).

The majority of the protein therapeutics are administered as solution through parenteral route by the end users [9]. However, to accommodate various requests from development, manufacturing and administration, the drug product presentation of therapeutic proteins can be in either solution or solid dosage formulations. Solution formulations can be administered directly to patients without preparation, whereas solid formulations typically require a reconstitution step before administration. Of the formulations that were approved for commercially available antibodies between 1986 and early 2021, 26.5% were in the form of lyophilized (freeze-dried) solids [9]. The solid dosage forms of drug products are selected especially when the protein is prone to degradation, aggregation or other chemical reactions in the solution. For example, at present all currently marketed antibody-drug conjugate drug products are in lyophilized form due to stability considerations [10, 11]. Particle counts for subvisible and visible particles in the reconstituted solution of dried solids are closely monitored and controlled during drug product development process.

Since 90 s, lyophilization has been developed for drying of therapeutic proteins [12–14]. Currently it remains to be the dominant drying method for biologic drug products. The reconstituted solution of lyophilized biologic drug products is generally of low level of visible and subvisible particles, meeting the requirement from USP. Particles in the reconstituted solution typically result from contamination rather than inherent protein aggregation. The in-process loss of proteins after lyophilization can be neglected. In recent years, drying technologies other than lyophilization have been attracting attention for their potential application in therapeutic biologics [15]. Spray-drying is among the most promising drying techniques for biologics, achieving higher drying efficiency than lyophilization and enabling particle engineering of the dried solids. It has been used for the manufacturing of an insulin product, Exubera, and a biologic fibrin sealant, Raplixa [16, 17]. Spray drying is considered as a more aggressive drying method compared to lyophilization. It applies high temperature on drug solutions and the droplets are exposed to air/liquid interface stress and shear stress in the drying process. The previous studies on the spray drying of proteins have focused on the level of protein aggregation/degradation after certain time of storage as dried powder, and how they compare to the corresponding lyophilized solids [18–20]. Nevertheless, protein stability during the reconstitution process of the spray-dried powder tends to be overlooked.

Initially, we observed the reconstituted solution of spray-dried protein powder was not clear and high level of visible particles can be identified. The formulation containing excipients (details not to be disclosed) has been commonly used for therapeutic proteins. In a separate study, the same formulation has been lyophilized and reconstituted in the same way as that of the spray-dried protein powder. The particle level in the reconstituted lyophilized solid was within the USP standard (data not shown). Thus, the observation of particle formation in the reconstituted solution is unique for the spray-dried powder of this formulation, which led us to further investigate the nature and the formation of these particles after reconstitution. To collect enough particles for analysis, formulation and spray drying process have not been optimized in the current study. Particle formation in the reconstituted solution can be observed visually. The particles below 200 μm were measured with microflow imaging (MFI). The insoluble particles were separated from the solution. Size exclusion chromatography (SEC) and differential scanning calorimetry (DSC) showed that the soluble protein in the reconstituted solution is comparable to that prior to spray drying. As suggested by Fourier transform infrared (FTIR) microscopy, the nature of the insoluble particle possessed protein structure. These insoluble particles were also referred to as insoluble aggregates. It has been challenging to analyze the insoluble aggregates, since they were not dry solids nor dissolved in solution. We have performed the hydrogen deuterium exchange (HDX) by comparing the deuterium uptake (D-uptake) differences between the supernatant and the aggregates to reveal the key structural changes for aggregate formation. A better understanding in the nature of the particle and the mechanism of protein aggregate formation could pave the way for the design of a spray-dry resilient protein construct and improvement of the robustness of the spray-drying process. This manuscript identifies a protein instability problem through the reconstitution of the spray-dried powder. The discussion on the formulation composition and spray drying process is not within the scope of this study.

## Materials and Methods

### Reagents and Materials

Two antigen-binding fragment (Fab) molecules, designated Fab-1 and Fab-2, were expressed and purified at AstraZeneca (Gaithersburg, MD). Deuterium oxide (D_2_O) (99.9% purity) was purchased from Cambridge Isotope Laboratory (Tewksbury, MA). Acetonitrile (liquid chromatography [LC]–mass spectrometry [MS] grade), DL-dithiothreitol (≥ 99.0%), and tris(hydroxymethyl)aminomethane (Trizma base; Sigma-Aldrich, St. Louis, MO) were purchased from Honeywell Research Chemicals (Charlotte, NC). Water (LC-MS grade), formic acid (LC-MS grade,  ≥ 99%) and phosphate-buffered saline (pH 7.4) were purchased from Thermo Fisher Scientific (Waltham, MA). Sterile water for injection (USP) was purchased from VWR (Radnor, PA). All other reagents were high-performance liquid chromatography (HPLC) purity grade and were purchased from Sigma-Aldrich unless otherwise specified.

### Spray-Drying of Fabs

The stock solution of each Fab was prepared with several excipients to reach a final concentration of 30 mg/mL, which was the same as that of the pre-spray drying solution. A placebo solution without Fabs and with the identical excipient component as the Fab solution was also prepared. Prior to spray-drying, the solution was filtered with a syringe-driver filter unit (0.2 μm). A Mini Spray Drier B-290 (Buchi, New Castle, DE) was used to generate spray-dried powders. The suboptimal spray-drying condition chosen here had the inlet drying temperature set at 125°C and the outlet temperature at 60–70°C. The feed rate of the solution was  ~ 1.5 mL/min, and the drying gas flow rate was  ~ 500 L/min. The spray-drying process was completed within 10–15 min. Optimization of the spray-drying process was considered to be outside the scope of this study and was not performed. After spray-drying, the powder was stored in clear glass vials before reconstitution.

### Particle Analysis by Microflow Imaging

Particles below 200 μm in the pre-spray drying solution and the reconstituted solution of the spray-dried powder at a concentration of 30 mg/mL were analyzed by microflow imaging (MFI) (ProteinSimple, San Jose, CA). The solutions were filtered before spray-drying, and the particle counts were close to 0 (≥ 5 μm,  ≤ 200 μm). Before each analysis run, a certain amount of the solution sample was purged though the analysis cell to optimize illumination. A total of 0.5 mL of each sample was analyzed with a purge volume of 0.2 mL. High particle counts in the reconstituted solution exceeded the detection limit of MFI. With limited spray-dried powder, a single sample was used for each measurement.

### Soluble Aggregate Analysis by Size Exclusion Chromatography (SEC)

The levels of soluble aggregates in the pre-spray drying solution and the reconstituted solution of the spray-dried powder were analyzed with SEC. Samples were either directly injected or diluted with phosphate-buffered saline, pH 6.8, to 10 mg/mL before injection. The mobile phase was sodium phosphate buffer at pH 6.8. The analysis was performed on a 1200 Series HPLC (Agilent, Santa Clara, CA) with a TSKgel™ G3000SWxl column (30 cm × 7.8 mm; Tosoh, King of Prussia, PA). Signal was collected by measurement of ultraviolet absorbance at a wavelength of 280 nm.

### Thermal Stability of Fab by Melting Temperature Measurement

Melting temperature (*T*_m_) of the Fab in the pre-spray drying solution and the supernatant of the reconstituted solution of the spray-dried powder were analyzed with capillary differential scanning calorimetry (DSC) (MicroCal VP-Capillary DSC; Malvern Panalytical, Malvern, UK). Samples were diluted to 1 mg/mL with the appropriate buffer. A total of 500 μL of solution was loaded in each well for analysis. The samples were heated from 20 °C to 100 °C at 1.5 °C/min. *T*_m_ values were determined with the built-in software.

### Insoluble Particle Analysis by Fourier Transform Infrared Microscopy

Fourier transform infrared microscopy (FTIR) was performed using a Spectrum 100 spectrometer with a Spotlight 400 microscope (PerkinElmer, Shelton, CT) to acquire the infrared spectra of the insoluble particles. The reconstituted solution of Fab-1 and Fab-2 was centrifuged, and the supernatant solution was removed. The sedimented particles on the bottom of the centrifuge tube were transferred to a clean glass slide and rinsed with filtered water to remove residual solution from the supernatant. The rinsed sample was transferred to a diamond compression cell, compressed, and examined with an EZ4D stereomicroscope (Leica, Deerfield, IL). The spectrum was collected using point mode at 2 cm^–1^ resolution in transmittance mode. Atmosphere correction was applied to exclude the effect of H_2_O and CO_2_.

### Aggregate and Soluble Protein Analyzed by Hydrogen–Deuterium Exchange

Soluble aggregation by hydrogen-deuterium exchange (HDX) has been reported previously [21–23]. To our knowledge, this is the first time that HDX has been used to study insoluble aggregates. To ensure that the same amount of insoluble aggregates was analyzed for each replicate and time point, a special sample treatment method was applied to prepare the insoluble aggregates for analysis. Spray-dried powders were reconstituted with 50 mM sodium phosphate buffer, pH 7.0, to reach a concentration of  ~ 10 mg/mL. The reconstituted sample was centrifuged at 13,000 × *g* for 5 min, and the supernatant was collected and used for the HDX experiment. The precipitation was washed 3 times by adding1 mL of 50 mM sodium phosphate buffer, pH 7.0, to the precipitate, which was then vortexed and centrifuged at 13,000 × *g* for 5 min; the supernatant was then removed, and this process was repeated twice, for a total of three times. The protein amount in the aggregate was estimated by comparing the initial protein amounts prior to spray-drying and the protein amounts in the supernatants of the reconstituted solutions. The same amount of protein was used in the aggregates and supernatant HDX experiments. The insoluble aggregate was vortexed briefly and pipetted to suspension, followed by manual transferring appropriate volume of suspension solution to each sample vial to keep the aggregates in suspension and maintain a constant amount of protein delivered in each replicate of each time point, since the automated workflow performed the HDX experiment over a 10 h period of time and the aggregates precipitated slowly to the bottom overtime, pre-dispensing the aggregates to the reaction vial was necessary.

HDX experimental procedures were automated and performed with an HDX Manager (Waters, Milford, MA), using a temperature-controlled liquid handling system (Trajan Scientific and Medical – LEAP, Morrisville, NC). Three sets of supernatant and insoluble aggregate HDX data were collected: Fab-1 at 24°C and 0°C, and Fab-2 at 24°C. For each HDX experiment, 3 μL of sample at a concentration of approximately 10 mg/mL was diluted tenfold with 50 mM sodium phosphate buffer, pH 7.0, in D_2_O to initiate HDX. All samples of aggregates were manually loaded onto the reaction vial to ensure protein aggregates are in suspension during this transfer and an equal amount of the insoluble aggregates was present in each reaction. The exchange reaction was maintained at 24°C or 0°C for various durations at 0.5, 1.5, 5, 15, and 60 min. The reaction was then quenched by the addition of 30 μL of pre-cooled quenching buffer, composed of 0.2 M glycine, 8 M guanidine HCl, and 0.5 M tris(2-carboxyethyl) phosphine at pH 2.5. The quenched samples were further diluted fourfold with 0.1% formic acid (pH 2.5, maintained at 0°C) and injected into the Waters HDX system. The samples were digested by passing through an online immobilized pepsin column (2.1 × 30 mm; Waters). The digested peptide mixture was trapped on a VanGuard precolumn (2.1 × 5 mm; Waters) for 2.5 min at a flow rate of 100 μL/min to desalt and then separated in a reversed-phase ultrahigh-performance LC column (Acquity BEH300 column, 1.0 × 100 mm; Waters) at a flow rate of 40 μL/min. The automation method was created and controlled in Chronos software (Trajan Scientific and Medical – LEAP, Morrisville, NC). Experiments were performed in duplicate for Fab-1 and in triplicate for Fab-2 due to different sample constraints.

### LC–MS Analysis of HDX Samples

The digested peptides were eluted with a 6.8-min gradient flow method (15–28% solvent B, acetonitrile with 0.1% formic acid; solvent A, H_2_O with 0.1% formic acid). For Fab-1, MS data were acquired on a Xevo G2-XS QTOF mass spectrometer (Waters) in positive electrospray ionization mode. For Fab-2, MS data were collected on a QExactive HF-X mass spectrometer (Thermo Fisher Scientific). The acquisition method was optimized for each instrument. For Fab-1, peptide identification was performed in sensitivity mode with a mass range of 300–1500 m/z for MS1, and data-independent acquisition (DIA) of tandem MS (MS/MS) by MS^E^, fragmented by collision-induced dissociation energy ramping from 25 to 50 eV. Lock mass was also collected using leucine-enkephalin. Labeled peptides were analyzed with the full MS scan and the lock mass without MS/MS. For Fab-2, peptide identification was performed on a Q Exactive HF mass spectrometer (Thermo Fisher Scientific) with the scan range of precursor ions set at 300–2,000 m/z for all samples and a resolving power of 120,000. The top 10 precursors with the highest intensities were selected for collision-induced dissociation in data-dependent acquisition (DDA) mode with a fixed collisional energy of 27% for fragmentation. Dynamic exclusion was activated for 5 s after each scan to enable MS/MS fragmentation of lower-abundance peptides. Deuterium labeling data were collected in a similar fashion without MS/MS scans. Xcalibur 2.2 (Thermo Fisher) and MassLynx 4.1 (Waters) software were used for data acquisition.

### HDX Data Analysis

Peptide identification was performed with Byos software (Protein Metrics, San Carlos, CA). The deconvoluted spectra were searched against the nonspecific digestion of amino acid sequences of Fab-1 and Fab-2. For Fab-1, because the MS/MS data were collected by MS^E^ in DIA mode, the searching parameters were set to allow up to 10 precursors in each MS/MS scan. For Fab-2, MS/MS data were collected in DDA mode, so the search allowed only one precursor for each MS/MS scan. The mass error tolerance window for the search was set to 10 ppm for all precursor ions and to 50 ppm for product spectra. The peptide list was imported into HDExaminer software (Sierra Analytics, Modesto, CA), and the numbers of deuterium uptake (D-uptake) of each peptide at different time points were calculated and later compared between the supernatant and the aggregates to generate the HDX differential plots.

### Protein Structure Modeling with Molecular Operating Environment Software

Homology models for Fab-1 and Fab-2 were built using the Molecular Operating Environment (MOE) antibody modeler [24, 25]. The MOE modeler used in this study was based on the Protein Data Bank [26, 27]. In Fab homology modeling, Fab-1 and Fab-2 sequences were searched against the database to identify collections of templates with the highest similarity for the framework region and complementarity-determining region (CDR) loops. The CDR loop templates were then grafted onto the light-chain and heavy-chain frameworks, followed by energy minimization in the transition area between CDRs and frameworks by AMBER-99 force field. HDX data visualization was performed with the HDX module of MOE. The color code scheme “Jet” was applied to visualize the D-uptake differences between the supernatants and the insoluble aggregates.

## Results

### Particle Levels in Reconstituted Spray-Dried Powder

**Fig. 1:**
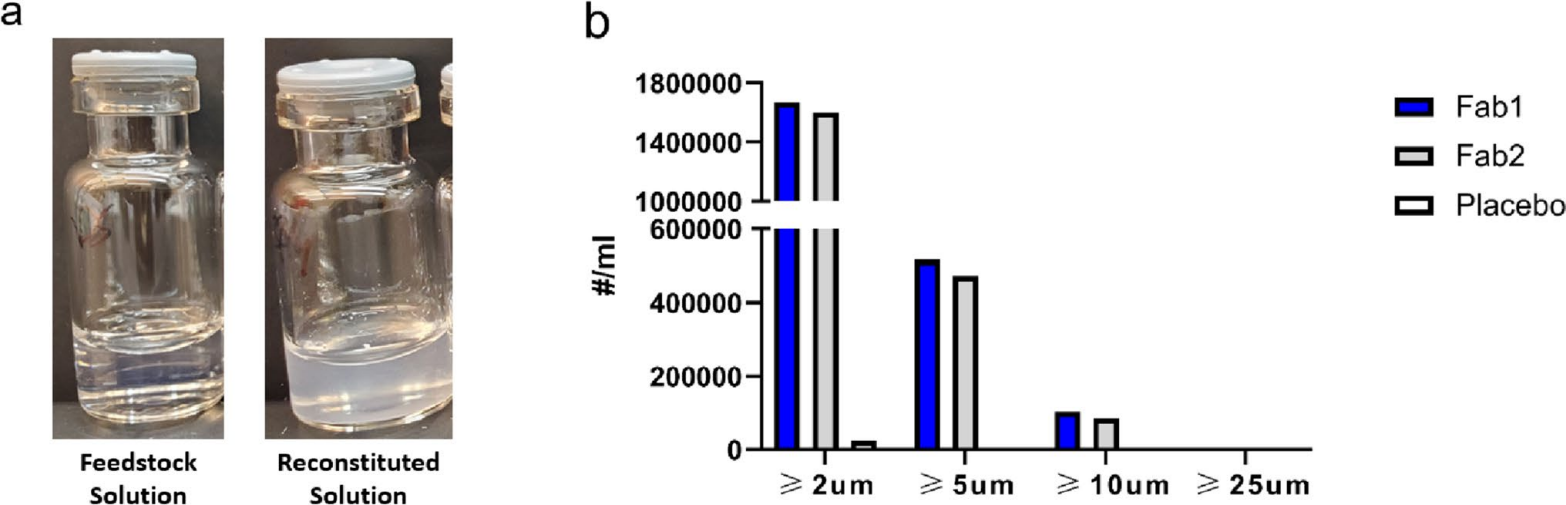
(**a**) Solution prior to spray-drying (left) and the reconstituted solution of spray-dried powder (right) of Fab-1. The pre-spray drying solution and the reconstituted solution of Fab-2 were similar to those of Fab-1 (not shown). The 2R clear glass vial holds 1 mL of solution. (**b**) Subvisible particles < 200 μm in diameter measured by MFI. The reconstituted solution of the spray-dried powder containing Fab-1, Fab-2, or placebo without protein was measured.

Spray-dried powders of Fab-1 and Fab-2 were prepared in clear glass vials and reconstituted with sterile water for injection (USP) to reach a final protein concentration of 30 mg/mL, which was the same as the concentration in the solution before spray-drying. The spray-dried powder from the placebo solution was reconstituted in the same way to reach the same excipient concentration with the Fab formulations. All the solutions were filtered to remove particles prior to spray-drying. After reconstitution, high levels of visible and subvisible particles were observed for both Fab-1 and Fab-2. The reconstituted solution of spray-dried powder was opaque, in contrast to the solution before spray-drying (Fig. 1a), owing to a large number of particles formed after reconstitution. Particles in the solution that were  < 200 μm were analyzed with MFI (Fig. 1b). The particle counts from the Fab formulations exceeded the detection limit of the MFI instrument. In contrast, no particles were observed in the reconstituted solution of the placebo spray-dried powder without the Fab, indicating that the particles formed after reconstitution were not a result of the undissolved excipients.

### Soluble Protein in Reconstituted Solution Compared with Protein in Solution Prior to Spray-Dry

**Fig. 2:**
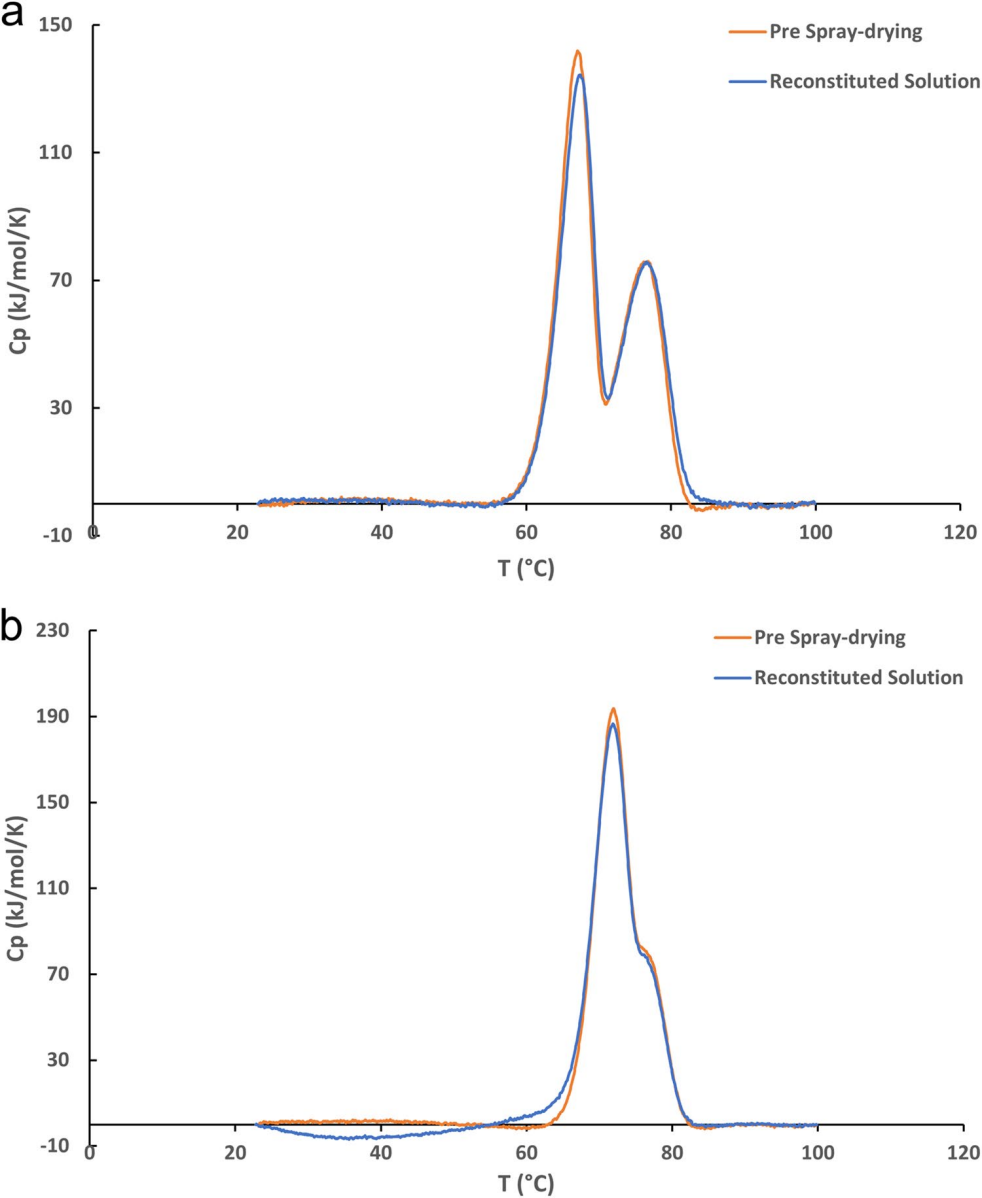
Thermal profile of the pre-spray drying solution and the reconstituted solution of Fab-1 (**a**), and Fab-2 (**b**) measured by capillary DSC.

Soluble aggregate levels and changes in *T*_m_ were analyzed and compared in the pre-spray drying solution and in the supernatant of the reconstituted solution of the spray-dried powder of Fab-1 and Fab-2 (Table I). For Fab-1, the difference in the percentage of aggregate between the pre-spray drying solution and the reconstituted solution supernatant was within 1%. Two distinct melting domains were observed in the thermal profile of Fab-1 (Fig. 2a) with no significant difference in *T*_m_ values for pre and post spray-drying. For Fab-2, the percentage of aggregate was almost identical in the pre-spray drying solution and in the reconstituted solution. Similar to Fab-1, no difference was found in the thermal profiles pre-spray drying solution and the reconstitution solution post spray-drying (Table I, Fig. 2b).

**Table I:** Soluble Aggregate, Monomer, and *T*_m_ of Fab-1 and Fab-2 in Pre-spray Drying Solution and Reconstituted Solution of Spray-Dried Powder Analyzed by SEC and Capillary DSC

Solution	Fab-1				Fab-2			
	Aggregate (%)	Monomer (%)	*T*_m_ 1 (°C)	*T*_m_ 2 (°C)	Aggregate (%)	Monomer (%)	*T*_m_ 1 (°C)	*T*_m_ 2 (°C)
Pre-spray dry	0.6	99.4	67.0	76.4	1.9	98.1	71.9	76.6
Reconstituted	1.3	98.7	67.4	76.6	2.0	98.0	71.9	76.5

DSC, differential scanning calorimetry; Fab, antibody fragment; SEC, size exclusion chromatography; *T*_m_, melting temperature

### Protein Signatures of Insoluble Particles by Infrared Microscopy

**Fig. 3:**
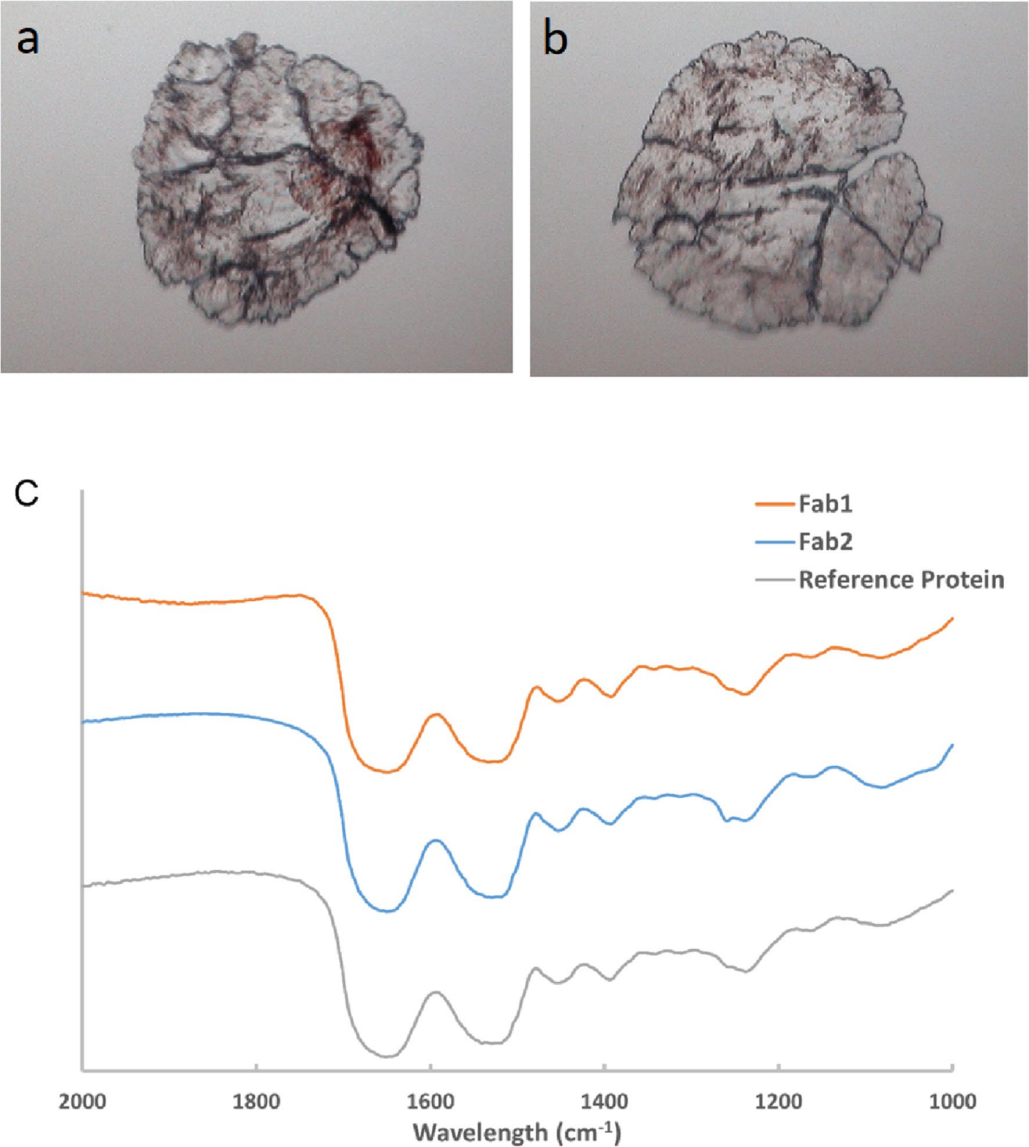
FTIR analysis of the insoluble particles from the reconstituted solution. Shown are the sediments of Fab-1 (**a**) and Fab-2 (**b**) after processing from the insoluble particles under FTIR. (**c**) FTIR spectra of the sediments of Fab-1 and Fab-2 compared with an internally manufactured reference protein. The FTIR spectra of the insoluble particles were comparable to that of the reference protein, indicating protein-like structures.

Particles in the reconstituted solutions of spray-dried powders were separated after centrifugation, removal of the supernatant, and rinsing with water. The resultant sediments were analyzed by FTIR to determine the nature of the insoluble particles. Images of the compressed sediments and the spectra of Fab-1 and Fab-2 revealed that the morphology of the sediment samples of Fab-1 and Fab-2 were similar (Fig. 3a, b). The distinctive protein features of the insoluble particles were indicated by the presence of amide I and amide II bands (1700–1500 cm^–1^ in the infrared spectra). The similarity between the reference protein spectrum and the infrared spectra of the insoluble particles confirmed that the insoluble particles were composed of protein. The FTIR results suggested that the nature of the insoluble particles formed after reconstitution of spray-dried powders were protein-like structures. The insoluble aggregates were further studied by HDX-MS.

### Aggregation Interface Revealed by HDX

To investigate the mechanism of aggregate formation and identify the interacting surface, HDX-MS was performed for Fab-1 and Fab-2, and the supernatant and the aggregate of the reconstituted spray-dried samples were compared. The precipitates collected by centrifugation remained insoluble during deuterium labeling but dissolved after mixing with the quench solution containing 8 M guanidine HCl. The dissolution of the insoluble aggregates in chaotropic agent indicated that the protein interaction in the aggregates was non-covalent. Dissolved aggregates enabled on-column pepsin digestion and peptide analysis by LC-MS. Successful HDX experiments require proteolytic digestion to yield enough common peptides from both the supernatant and the aggregate to localize conformational differences between the two. The sequence coverage for Fab-1 was 96% for the heavy chain with 3.2 average redundancy and 99% for the light chain with 3.4 average redundancy. For Fab-2, heavy-chain sequence coverage was 93.5% with 3.9 redundancy and light-chain coverage was 100% with 5.3 redundancy.

**Fig. 4:**
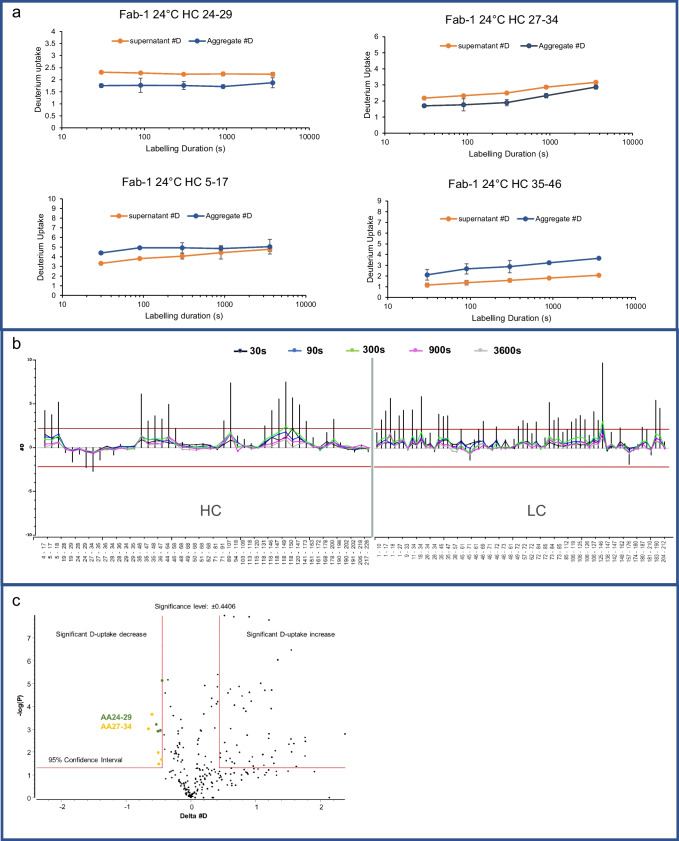
HDX results for comparison of Fab-1 supernatant and aggregate at 24°C. (**a**) D-uptake plots (measurements in duplicate) indicated that two peptides in heavy-chain CDR-1 are critical regions for aggregate formation. The supernatant (red) compared with the aggregates (blue) show D-uptake reduction in heavy-chain CDR-1 peptides AA24–29 and AA27–34, suggesting that Fab-1 formed aggregates through heavy-chain CDR-1. AA5–17 and AA35–46 exhibited increases in D-uptake, suggesting that these regions in the aggregates are more dynamic. (**b**) HDX differential plot of Fab-1 light and heavy chains shows the difference in D-uptake in the supernatant and the aggregate. Vertical lines represent the total difference in D-uptake for each peptide from five time points. The red lines represent the significance criteria for the sum of five labeling time points determined by HDExaminer, calculated by including the variance in the replicate experiments. (**c**) Volcano plot of all time points of all peptides. The negative log of the *P* value is shown on the y-axis. Each time point of a peptide is plotted by the D-uptake difference and the *P* value of that time point from the *t* test. The vertical lines indicate significance levels of the difference from the data variance in replicate experiments. The red horizontal line represents 95% confidence interval, and the vertical lines indicate the significance level of differences. Data points at the upper left exhibited significant protection, and those at the upper right were significantly more dynamic. The only significant protections are data points from peptides AA24–29 and AA27–34.

Greater D-uptake usually indicates a more disordered structure than in the region with less D-uptake. When the HDX profile of the Fab-1 supernatant was compared with that of the aggregate, extensive increases in D-uptake were observed in various regions of both the heavy chain and the light chain. The increase was more prominent in the heavy chain than in the light chain and was greater in the variable domain than in the constant domain (Fig. 4b). The framework regions surrounding heavy-chain CDR-1, namely, the N-terminus and AA35–46, exhibited the highest increase in D-uptake, indicating that this region undergoes vigorous conformational changes in the spray-drying process and becomes much more structurally dynamic. In contrast, the AA24–34 region, which included heavy-chain CDR-1, showed protection in insoluble Fab-1, in contrast to the soluble form. The D-uptake of peptides AA24–29 and AA27–34 in heavy-chain CDR-1 decreased across all time points (Fig. 4a).

The statistical significance of the D-uptake differences was evaluated and plotted by volcano plot (Fig. 4c). The X-axis of the volcano plot is the difference in D-uptake between the insoluble aggregate and the soluble protein. A negative difference indicates more protection, whereas a positive difference suggests a more dynamic structure in the insoluble aggregate than in the soluble protein. The negative log of the *P* value is shown on the y-axis of Fig. 4c. For Fab-1, AA24–29 and AA27–34 showed significant protection, exceeding both criteria that the differences should be beyond the 95% confidence interval and the significance threshold calculated by assessing the experimental variance of all observed peptides. The peptides shown in the upper-right area of the volcano plot were more dynamic in the aggregate than in the supernatant. Peptides AA5–18 and several peptides in the framework region between heavy-chain CDR-1 and CDR-2, which are immediately adjacent in sequence to heavy-chain CDR-1, were more structurally dynamic in the aggregated protein.

HDX with Fab-1 at 0°C was also performed in an attempt to interrogate the structural dynamics of the fast-exchanging residues [28]. As the reaction temperature decreases, the HDX kinetics slow down. If any of the fast-exchanging residues are involved in aggregate formation, we would observe D-uptake differences between the soluble protein and the aggregate on these residues. Interestingly, HDX labeling at 0°C identified the same region to be more protected in the aggregate than the labeling at 24°C (Figure S1). More regions exhibited increased D-uptake at 0°C than at 24°C, indicating that the conformational changes upon forming aggregates at 0°C was more extensive than that observed in the 24°C labeling experiment (Figure S1). Comparing D-uptake profiles for 24°C labelling (Fig. 4a) with 0°C labelling (Figure S1a), in both supernatant and aggregates, D-uptake is higher in 24°C labelling than 0°C labelling, however, the aggregates and supernatant difference is greater in 0°C labelling than in 24°C labelling. Since the exact same quenching, digestion, and separation procedures were performed in 0°C and 24°C labelling, it is very unlikely that experimental artifacts, i.e., differences in back-exchanges, is the main reason for this observation. The more extensive increases in D-uptake for 0°C labelling can be attributed to slower exchanging residues becoming more solvent exposed in the aggregates due to conformational changes induced by spray-drying. The protein structure was likely to be more dynamic and to form precipitates by non-covalent binding through heavy-chain CDR-1.

**Fig. 5:**
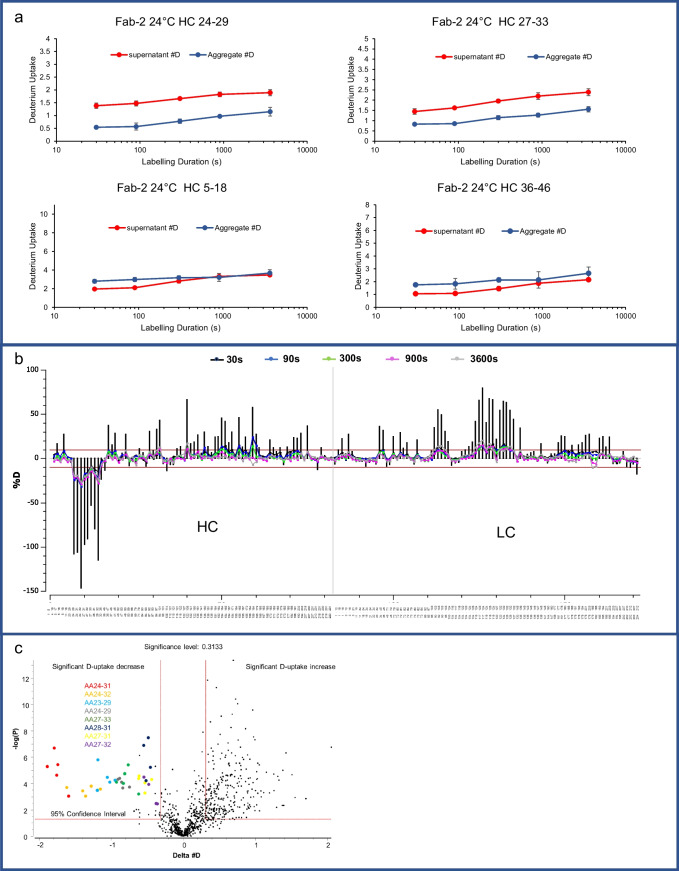
HDX results for comparison of the Fab-2 supernatant and aggregates at 24°C exchange temperature. (**a**) D-uptake plots (measurements in duplicate) of two peptides in heavy-chain CDR-1 showed protection in the aggregates. The supernatant (red) compared with the aggregate (blue) showed D-uptake reduction in AA24–29 and AA27–33, which are heavy-chain CDR-1 peptides, suggesting that the protein formed aggregates through this region. AA5–18 and AA36–46 exhibited increases in D-uptake, suggesting that these regions in the aggregates were more dynamic. (**b**) HDX differential plot of Fab-2 light and heavy chains shows the difference in D-uptake between the supernatant and the aggregates. Heavy-chain CDR-1 exhibited significant protection, and various framework regions and constant domains were unfolding. (**c**) Volcano plot of all time points of all peptides. Data for heavy-chain CDR-1, shown at the upper left, indicated significant protection.

HDX was used to investigate the mechanism of aggregate formation for Fab-2 by comparing the structural dynamics between the soluble protein and the aggregate. Two key findings in the Fab-1 HDX analysis were consistent for Fab-2: (1) various regions showed higher D-uptake in the aggregate than in the supernatant (Fig. 5b), and (2) AA23–33, the identical region as in Fab-1, showed significantly more protection in the Fab-2 aggregate (Fig. 5a, c). Although Fab-1 and Fab-2 were designed for different targets and therefore have different CDRs, their framework regions and constant domains were highly similar. Fab-1 and Fab-2 were expected to undergo similar structural changes when exposed to stresses during the spray-drying process. Heavy-chain CDR-1 in Fab-1 and Fab-2 had high sequence similarity. Specifically, AA24–29, which exhibited significant protection in both the Fab-1 and the Fab-2 aggregates, had the identical sequence in both Fab-1 and Fab-2 and contained several hydrophobic residues. Thus, heavy-chain CDR-1 was identified as the key region for forming aggregates and precipitation in Fab-1 and Fab-2, most likely through hydrophobic interaction manifested by the richness of hydrophobic residues in this segment.

Several possible aggregation mechanisms of therapeutic proteins were observed and investigated [1]. It’s established that for nonnative polymerization, structure changes and formation of the “reactive” monomers are in the first stage of aggregation formation. In the second stage, reversible oligomers form, followed by nucleation in the third and fourth stage through rearrangements or realignment. In the final stage, aggregates form soluble or insoluble polymers by monomer addition or cluster-cluster aggregation. Our HDX data showed hydrophobic HC-CDR-1 is protected in the aggregates, suggests it is a specific interaction between the protein monomers and majority of the protein population is bounded through this region in the aggregates. Thus, HC-CDR-1is considered to be the important reactive site contributing to forming the initial oligomers and is the key for nucleation. Due to the extensive increases in the aggregate D-uptake, less significant protections were not observed by HDX. It’s possible that these interactions with multiple less specific interaction sites were involved in the following aggregation stages.

### Structural Dynamics of Fab Molecules by HDX

The crystal structures of the Fab fragments are well established [29]. In the variable domains of Fab-1 and Fab-2, the framework regions formed two antiparallel beta sheets connected by three loops of CDRs on the outside that were structurally dynamic to enable antigen binding. The constant domain had similar structural elements as the variable domain, which was also composed of antiparallel beta sheets, further stabilizing the protein structure.

The secondary structure of the Fab-1 homology model was compared with the HDX kinetics (Figure S2). The loops generally showed faster D-uptake than the beta sheet regions, indicating that the loops had greater solvent accessibility and faster conformational dynamics. The N-terminal region of the heavy and light chains of Fab-1 also showed faster HDX kinetics, as they were less structured and more solvent accessible. Among the CDRs, heavy-chain CDR-1 and light-chain CDR-2 were the most conformationally dynamic regions in Fab-1 (Figure S2a). The HDX results for Fab-2 were also mostly consistent with the secondary structure of the homology model, as the loops exchanged faster than the beta sheets or the alpha helices (Figure S2b). The N- and C-termini had higher D-uptake due to higher solvent accessibility. Among the CDRs, heavy-chain CDR-1 and CDR-3 were the more conformationally dynamic regions in Fab-2. In many cases, residues in heavy-chain CDR-3 region are responsible for target binding. Fab-2 heavy-chain CDR-3 was very rich in aromatic and aliphatic residues, so it’s conceivable that Fab-2 bind the target by hydrophobic interaction through these residues. To facilitate such interaction, heavy-chain CDR-3 was structurally dynamic and solvent accessible. Comparing with Fab-2, Fab-1 heavy chain CDR-3 loop is shorter and less dynamic, since Fab-1 and Fab-2 are designed to bind different targets, structural differences in their CDRs are expected. Although heavy-chain CDR-1 was also rich in aromatic residues, evidently it did not directly participate in target binding.

## Discussion

Lyophilization, or freeze-drying, has been applied in the pharmaceutical industry as the drying method for biologics since the early 1990s [30–32], as solid dosage forms offer many benefits such as longer shelf-life and reduced shipping costs. Meanwhile, there is an increasing interest in the application of drying technologies that are alternatives to lyophilization for biologics [33, 34]. These efforts have typically focused on the development of drying technologies that are more efficient and can be used to control particle size and shape [35, 36]. Spray-drying has long been used for the drying of small-molecule drugs. Over the years, several studies have been undertaken to overcome the challenges of spray-drying of biologics [18, 19, 37–39]. Because biologic drug products are usually administered through parenteral route, it is important to monitor particle formation after reconstitution of the dried solids. It has rarely been seen as an issue for lyophilized drug products. Unexpectedly, we have observed high amount of particle formation in the reconstituted solution of freshly spray-dried protein powder.

We investigated two Fab molecules (Fab-1 and Fab-2) and found the reconstituted solution exhibited a high level of particles  < 200 μm in diameter (Fig. 1) and the particles were generated from the protein rather than the excipients (Figs. 1b and 3). Soluble aggregation levels and thermal stability were comparable for the protein in solution before and after spray-drying (Table I and Fig. 2). These particles were referred to as insoluble aggregates in this study.

The formation of insoluble aggregates was further studied by HDX analysis. The insoluble aggregates were isolated from the supernatant of reconstituted solution. HDX experiments were performed on both the insoluble aggregates after ultracentrifugation and the supernatant of the reconstituted solutions. Similar investigations on insoluble aggregations of amyloid fibrils were performed previously [40, 41]. Due to solubility challenges, D-uptake data with spatial resolution was obtained by NMR, and LC–MS analysis provided global D-uptake for the protein. Several studies on amyloid fibrils reported two distinct isotopic envelopes from the intact analysis of the deuterated aggregates, indicating the aggregates contain a solvent inaccessible core and the exchange kinetics falls under Ex1 regime. Ex1 describes the phenomenon when the rate of protein closing is much slower than the intrinsic exchange rate and give rise to multiple isotopic envelopes in the peptide spectra, whose intensity ratios are dependent on the exchange time. Giving the exchange process took longer than 4 days, which was slower than the common exchange rates observed for native proteins, it is conceivable that the exchange kinetics of amyloid fibril fall under Ex1 regime. However, for Fab-1 and Fab-2 aggregates, which do not form insoluble aggregates under the native state but rather in a more dynamic conformation, exchange in a much faster rate, and the deuterated spectra do not show isotopic envelopes consistent with Ex1 kinetics. Our results indicate the majority of the protein population was in a more dynamic conformation and is compatible with Ex2 exchange kinetics since Ex2 describes the exchange kinetics for protein in an open conformation and the exchange rate is the intrinsic exchange rate or when protein is in the stable closed conformation and the rate of protein closing is much greater than the intrinsic exchange rate.

Protein self-association was also studied previously [42]. As protein concentration increases, protein self-association increases manifested by the higher viscosity in solution. The HDX experiments comparing the D-uptake of low and high concentration protein samples revealed regions been protected in high concentration condition, as well as regions exhibiting higher structural dynamics. These increases observed were attributed to allosteric interactions induced by the distant dynamic coupling effect. Despite of these changes, most of the protein structure remains intact in its native state, which is very different from our observation for the aggregates. Our experiments result showing extensive denaturation of the native structure is the key for forming the insoluble aggregates.

Comparing the HDX results for the aggregate and the supernatant, their differences in D-uptake reveal the protected region in the insoluble aggregate, which is potentially the binding interface for forming the initial aggregates. In Fab-1 and Fab-2, similar segments in the framework and constant regions had higher D-uptake in the aggregate than in the supernatant. Moreover, the AA23–33 sequence in heavy-chain CDR-1 in both Fabs showed significantly more protection in the aggregates. HDX heat maps (Figure S2) suggest that heavy-chain CDR-1 was one of the most conformationally dynamic regions in the native Fab-1 (Figure S2a) and Fab-2 (Figure S2b). It’s rich in hydrophobic residues but was not directly involved in target binding. Upon forming aggregates, heavy-chain CDR-1 in the AA24–34 region showed significant protection, suggesting that the hydrophobic interaction at the binding site was critical for aggregate formation (Figs. 4 and 5). In contrast, the framework regions, such as AA35–46, became more conformationally dynamic in the aggregates (Figs. 4 and 5). The CDRs were connected and were displayed by the structured framework regions that forms the anti-parallel beta sheets in the native state. These regions were structurally stable in the native form but could have lost structural integrity and partially unfolded after spray-drying.

**Fig. 6:**
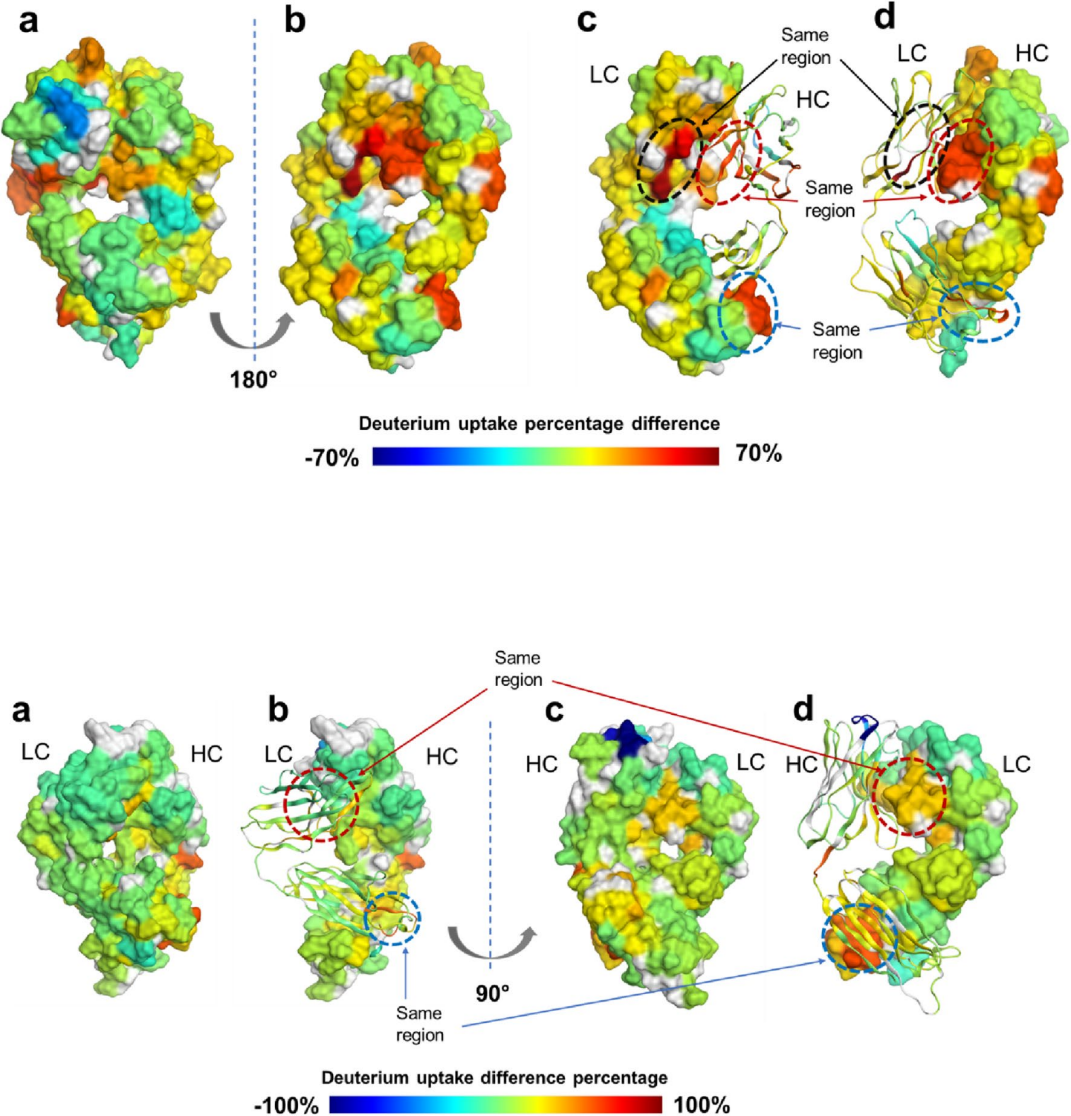
Difference in D-uptake percentage between supernatant and aggregate, calculated as (% D-uptake difference) = [(% D-uptake of Fab aggregate) – (% D-uptake of Fab supernatant)]. Top: Difference in percentage of D-uptake in the supernatant and the aggregates for Fab-1. (**a**) Heavy-chain CDR-1 is shown in blue due to its decrease in D-uptake, indicating that it was protected in the aggregates. (**b**) One hundred eighty–degree rotation of the view shown in (**a**); regions shown in red represent structural unfolding. (**c**) Same view as (**b**), but the heavy chain is shown so as to view the relative positions of unfolding regions on the light chain. (**d**) Same view as (**b**) with the light chain shown as a ribbon to exhibit unfolding regions on the heavy chain. The same unfolding regions shown in (**c**) and (**d**) are indicated with arrows; these regions were mostly on the contacting surface between the heavy chain and the light chain, indicating that they may have undergone dissociation in the aggregates. Bottom: Difference in D-uptake between the supernatant and the aggregates for Fab-2. Heavy-chain CDR-1 is shown in blue to indicate that it was protected in the aggregates, similar to Fab-1. The red area represents structural unfolding regions. (**a**) and (**b**) are the same view, but the light chain is shown as a ribbon in (**b**) to show the unfolding regions on the heavy chain. The view shown in (**b**) is the same as the view for (**c**) and (**d**), rotated 90 degrees; (**d**) shows the heavy chain as a ribbon to illustrate the unfolding regions on the light chain. The unfolding regions in the dotted circles are the same regions in (**b**) and (**d**); they were mainly on the contacting surface of the heavy chain and the light chain, indicating that Fab-2 aggregates also underwent dissociation of the heavy chain and the light chain.

**Fig. 7:**
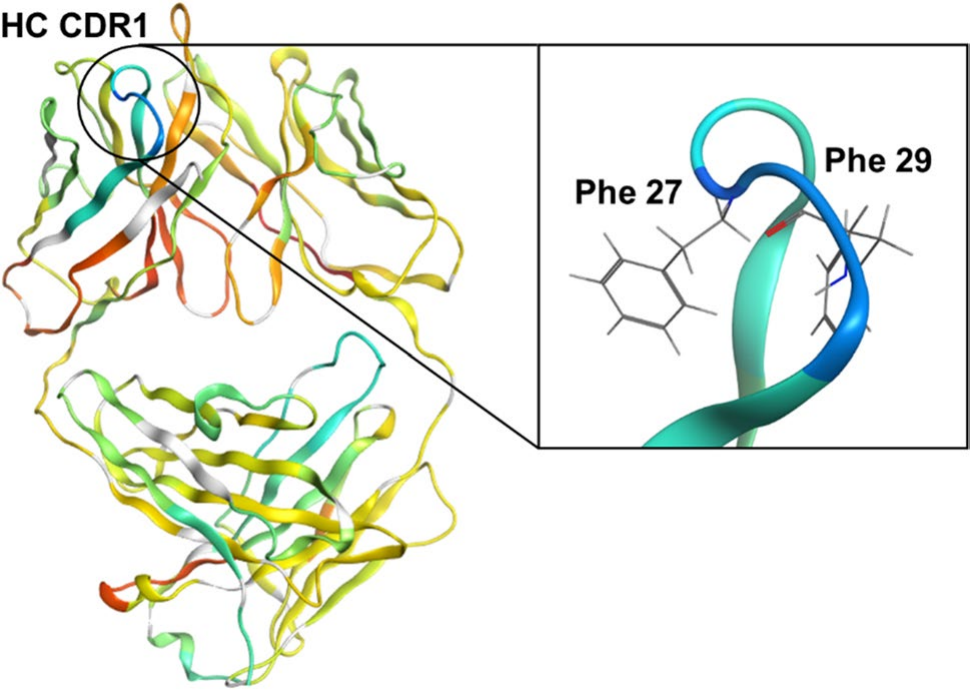
Enlarged view of heavy-chain CDR-1 of the Fab-1 homology model, which contained two Phe with their side chains oriented toward the inside. During structural unfolding, as constraints are removed, these residues may become more dynamic and are followed by changes in side-chain orientation. Binding with other Fab molecules could occur through the hydrophobic interactions with exposed Phe.

To enable visualization of the conformational changes, D-uptake differences on the surfaces of the Fab-1 and Fab-2 homology models are color-coded in Fig. 6. Blue regions in the figure indicate decreased D-uptake from the supernatant to the aggregate, suggesting increased protection in the aggregates. Red areas represent increased D-uptake, most likely as a result of partial structural unfolding in the aggregates. In the three-dimensional structure shown in Fig. 6, the only heavy-chain CDR-1 in the blue region is on the molecule surface but is enclosed by heavy-chain CDR-2 and CDR-3 and the framework regions. Most of the unfolded region is located at the interface between the heavy chain and the light chain (Fig. 6). This suggests that the hydrophobic interaction between the heavy chain and the light chain could have been disrupted despite the fact that disulfide bonding at the C-terminus covalently connects them. The loss in structural integrity could lead to the deformation of the protein’s higher-order structure. As a result, the loop region in heavy-chain CDR-1 was more conformationally dynamic, and a previously buried Phe side chain in the structure of the native protein was exposed (Fig. 7), which accelerated aggregation through hydrophobic protein-protein interactions. Another possible factor contributing to the formation of insoluble aggregates was the heavy-chain and light-chain dissociation removes the structural hinderance, enabling the formation of protein oligomers binding through the heavy-chain CDR-1. Increasing number of oligomer formation led to subsequent growth in aggregate size and changes in morphology.

Aggregation is usually governed by complex pathways of protein-protein interactions. The stresses from the spray-drying process such as high heat, interfacial stress and shear stress could have uneven impacts on the protein structures depending on the stress conditions they experienced. The structures of the protein in spray-dried powder could be more heterogeneous compared to those in lyophilized solids [43], which is more susceptible to instability. For a subset of molecules, the interactions between the heavy and light chains and the higher-order structure were disrupted. Structural constraints at heavy-chain CDR-1 were disrupted, exposing hydrophobic residues. Simultaneously, structural hinderance was removed as heavy-chain and light-chain dissociated, enabling oligomer binding through heavy-chain CDR-1. After reconstitution with water, the protein fraction with disrupted structure and/or heavy-light chain interactions led to the initial formation of oligomers. These oligomers could then rearrange their structure to further stabilize the interaction, resulting in irreversible aggregates. Numerous aggregation reactions can happen simultaneously to eventually form visible and subvisible particles in the reconstituted solution.

Several factors could have potentially contributed to the particle formation in the reconstituted solution of the spray-dried protein powder. Spray drying is considered as a more aggressive drying method than lyophilization, with the usage of high temperature in its drying process [34]. More heterogeneous protein structure can result from the aggressive drying process [43], which could lead to the initial protein–protein interactions followed by the formation of dimer and trimer [3]. These reactions can quickly happen upon the moment of the spray-dried powder in contact with the reconstitution solution. Additionally, protein tends to show structure heterogeneity when distributed on the surface of the spray-dried particles [44]. The protein tends to be more stable when there is higher portion distributed inside the powder particle then on the surface[44]. It has been shown lowering of the specific surface area of the powder can stabilize protein structure [45, 46]. Lastly, the protein can be engineered to alter the HC-CDR-1 sequence and reduce the protein-protein interaction. The aggregation behavior and overall stability will be assessed for these engineered protein constructs. The optimized construct with low aggregation, improved stability, and unreduced activity will be selected and further developed.

## Conclusions

This study provides an in-depth understanding of insoluble aggregate formation after the reconstitution of spray-dried protein powders. Two Fab molecules were formulated, spray-dried, and reconstituted. High levels of particles were observed after reconstitution of the protein powders that had been spray-dried under suboptimal conditions. Levels of soluble aggregate and *T*_m_ confirmed that the protein remained in its native state in the soluble fraction of the reconstituted solution. The insoluble particles were collected from the reconstituted solution, and FTIR showed that the particles were protein related, which was also confirmed by HDX analysis. The HDX results revealed the mechanism of aggregate formation for the insoluble aggregate, suggesting that stresses from spray-drying disrupted protein structure and exposed the hydrophobic residues in heavy-chain CDR-1, and insoluble aggregate formed from the proteins with disrupted structure through hydrophobic interactions upon reconstitution with water. These results can contribute to efforts to design spray-dry resilient protein constructs and improve the robustness of the spray-drying process.

### Supplementary Information

Below is the link to the electronic supplementary material.

Supplementary file1 (DOCX 389 KB)

## Data Availability

The data that support the findings of this study are available on request from the corresponding author, YT. The data are not publicly available due to company restriction.
