# Supplementary material for: Mechanism of Insoluble Aggregate Formation in a Reconstituted Solution of Spray-Dried Protein Powder
Source: Pharm Res. 2023 May 2;40(10):2355–70. doi: 10.1007/s11095-023-03524-x (PMC10661820; doi:10.1007/s11095-023-03524-x)
Supplement: Supplementary file 1 — Supplementary file1 (DOCX 389 KB) [file 11095_2023_3524_MOESM1_ESM.docx]

**Mechanism of Insoluble Aggregate Formation in a Reconstituted Solution of Spray-Dried Protein Powder**

Tao Y, Chen Y, Howard W, Ibrahim M, Patel SM, McMahon WP, Kim YJ,

Delmar JA, Davis D

**Supplemental Information**

**Figure S1.** HDX results for comparison of Fab-1 supernatant and aggregates at 0°C. (a) Deuterium uptake (D-uptake) plots (measurements in duplicate) indicates that two peptides in heavy-chain complementary-determining region 1 (CDR-1) are critical regions for aggregation formation. The supernatant (red) compared with the aggregates (blue) show D-uptake reduction in AA24–28 and AA24–29, suggesting that the protein formed aggregates through this region. AA5–17 and AA35–46 exhibited increased D-uptake, suggesting that these regions in aggregates are more dynamic. (b) Hydrogen-deuterium exchange (HDX) differential plot of antibody fragment 1 (Fab-1) light chain and heavy chain shows the difference in D-uptake in the supernatant and the aggregates at 0°C. Results similar to those of HDX at 24°C were observed, indicating that heavy-chain CDR1 had significant protection and framework regions and constant domains were more dynamic in the aggregates.


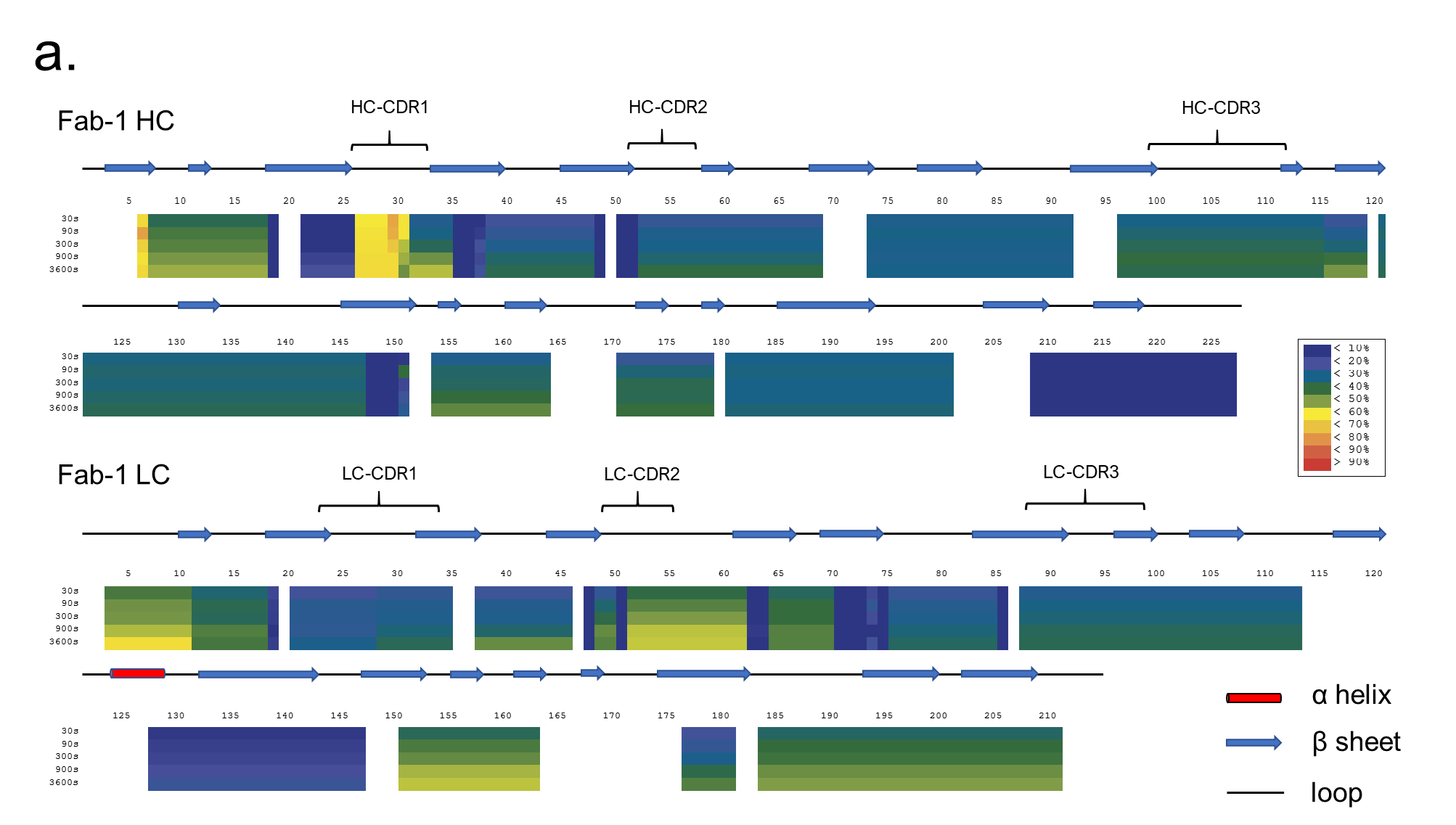


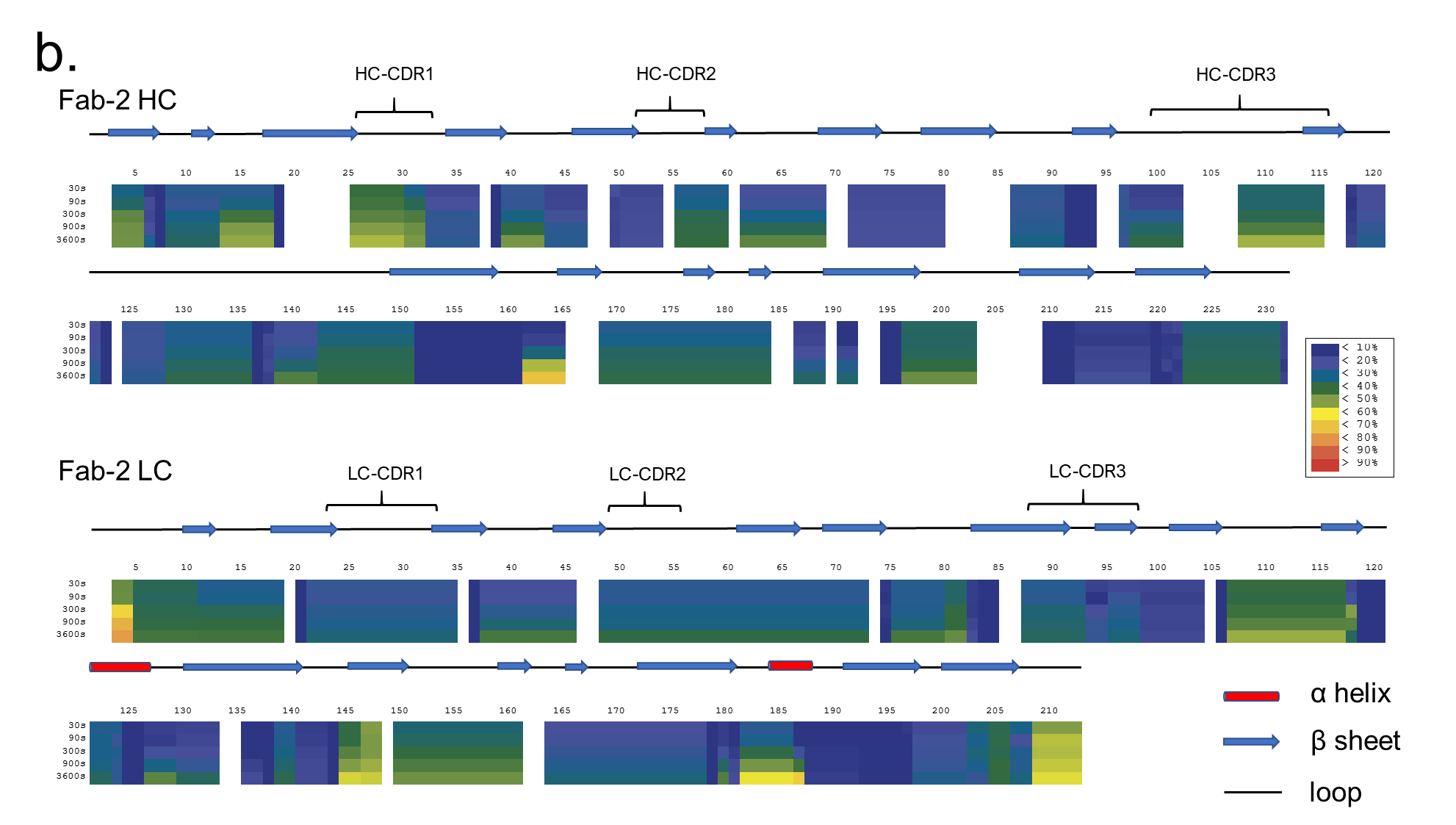


**Figure S2.** HDX heat map for Fab-1 (a) and Fab-2 (b) supernatant. D-uptake was measured at 0.5, 1.5, 5, 15, and 60 min, and its level at time each point is colored on to the sequence by HDExaminer, which takes overlapping peptides into account. The secondary structure from the protein homology model is labeled on top of the heat map, in which red cylinders represent alpha helices, blue arrows indicate beta sheets, and black lines are the loops. CDRs are indicated on top.
